# Beyond the pandemic: rising administrative demands and changing disease profiles in primary care

**DOI:** 10.1186/s13584-025-00707-2

**Published:** 2025-07-29

**Authors:** Avivit Golan Cohen, Shlomo Vinker, Eugene Merzon, Ilan Green, Ariel Israel

**Affiliations:** 1 Department of Family Medicine, Faculty of Medicine, Tel Aviv University, 6910127 Tel Aviv, Israel; 2 Leumit Health Services, 23 Shprinzak St., 6473817 Tel Aviv, Israel; 3 Adelson School of Medicine, Ariel University, 4077601 Ariel, Israel; 4 Department of Epidemiology and Disease Prevention, School of Public Health, Tel Aviv University, 6910127 Tel Aviv, Israel

**Keywords:** Primary care, Reasons for visits, Workload, COVID, ICD-9 codes

## Abstract

**Background:**

The COVID-19 pandemic has transformed healthcare, affecting the diagnosis and management of common diseases. Our study aimed to assess the effect of the changes in reasons for primary care visits on primary care physicians’ (PCPs’) workload from 2019 to 2023, focusing on non-COVID-related diseases.

**Methods:**

A cross-sectional study of electronic medical records conducted at Leumit Health Services between 2019 and 2023, approximately 510,000 patients who had at least one consultation with a PCP were included. The study categorized visits using ICD-9 codes and calculated the number of visits and the accumulated annual duration of time (AADT) for each code group.

**Results:**

In 2023, there was a significant 38.9% increase in administrative visits compared to 2019, with these visits accounting for 21.8% of AADT. Additionally, a consistent rise in visits for hyperlipidemia, obesity, and diabetes was noted. Conversely, the AADT for respiratory tract infections and sexually transmitted diseases markedly declined. A lesser, yet still notable, decrease was observed in other infectious diseases, injuries, heart diseases, and pulmonary diseases.

**Conclusions:**

COVID-19 altered the distribution of primary care visit reasons and subsequently impacted the burden on PCPs. Notably, there was an increase in visits for bureaucratic issues and a concerning reduction in follow-ups for cardiovascular risk factors, alongside a rise in metabolic conditions. These trends persisted even after the pandemic waned, despite the removal of social restrictions. Policymakers should evaluate how to optimize the utilization of PCPs’ time and explore methods to regulate demand for improved efficiency.

**Supplementary Information:**

The online version contains supplementary material available at 10.1186/s13584-025-00707-2.

## Background

The COVID-19 pandemic prompted adaptive responses from primary care physicians (PCPs) worldwide, leading to significant transformations in community health care management [1–3]. These changes included a reduction in ambulatory services [4], implementation of preventative measures for at-risk populations, adoption of new technologies for healthcare service accessibility [5], and delegation of care responsibilities to other healthcare staff [6, 7]. Consequently, there was a pronounced decline in face-to-face (FTF) visits and a notable increase in telemedicine visits [8]. These shifts occurred amidst rising COVID-19 rates, lifestyle changes due to social distancing mandates, and increased stress due to health concerns and detrimental financial effects. In Israel, the peak of the COVID-19 pandemic occurred in mid-January 2022, followed by significantly lower waves of morbidity in the subsequent years up to the present. Restrictions aimed at reducing infection were gradually lifted starting at the end of 2021, with complete removal by March 2022.

Despite its importance, particularly during the COVID-19 pandemic, there exists a significant research gap concerning the dynamics of disease prevalence and primary care workload. Understanding this data is crucial for guiding medical services, developing targeted solutions to address evolving healthcare needs, and improving preparedness for future challenges. This has significant implications for social, economic, and public health aspects [9].

Pre-pandemic literature highlights diverse patterns across countries, lacking standardized reporting approaches. For instance, a 2018 review of 18 studies from 12 countries identified prevalent reasons for visits such as upper respiratory tract infections, hypertension, routine health maintenance, and mental health conditions [10]. A 2016 German study disclosed that administrative visits constituted 54.3% of all visits, with the top non-administrative reasons being cough, back pain, shoulder problems, knee problems, and shortness of breath [11]. Limited data from Israel indicates that the most prevalent acute diseases include respiratory and digestive tract infections, as well as musculoskeletal issues. Chronic diseases primarily consist of hypertension and diabetes management, with administrative visits accounting for 15%, while preventative medicine visits comprised only 5% [12].

Initial observations from the pandemic’s onset in 2020 indicate a substantial increase (from 19 to 42%) in visits diagnosing anxiety or depression, alongside a marked decrease (from 47 to 86%) in diagnoses related to coughs and colds [5, 8].

Additionally, 2020 witnessed an approximately 40% reduction in new diagnoses including cardiovascular risk factors, chronic diseases, injuries, respiratory infections, and certain tumors [5, 13, 14]. Conversely, visits related to socioeconomic and housing problems increased [15].

The limited information published on this topic post-2020 constrains the ability to evaluate the impact of lifting restrictions, the extent of return to routine behaviors, the changes in disease characteristics, and the necessity for corrective actions to mitigate emerging risks.

Additionally, there is a lack of data on how changes have affected the burden of various diseases on PCPs. Current reports offer solely the number of visits per diagnosis, overlooking potential shifts in morbidity complexity and time investment. Relying solely on the number of visits for workload assessment may also lead to potentially misleading conclusions, as it ignores variations in time investment influenced by factors such as individual characteristics, treatment protocols, and payment methods. Furthermore, the influence of synchronous and asynchronous telemedicine visits on time consumption is not considered, despite their known effect on visit length.

To address limitations associated with traditional metrics, our study employs the Accumulated Annual Duration of Time (AADT) measure, which aggregates a patient’s total minutes spent with a PCP over a year. AADT, as demonstrated by Nathan et al., offers a method of assessing time investment, and enables comparisons between healthcare systems [16].

Our objective was to evaluate the shifts in PCPs’ workload related to non-COVID-19 diseases from 2019 to 2023. The study duration allowed for an analysis of diagnosis distribution during peak morbidity and a comparable period post-decline, providing insights into trends over time. For comprehensive presentation, the entire year of 2023 was included, despite Israel being at war in the last quarter.

## Methods

*Study design and sampling strategy*: A cross-sectional study design leveraging electronic medical records of all patient visits to PCPs at Leumit Health Services (LHS) between 2019 and 2023.

*Setting*: LHS is one of Israel’s four health maintenance organizations (HMOs), insuring approximately 710,000 citizens with eligibility for all offered services. Each member is assigned a PCP but can consult another PCP if the assigned one is unavailable. LHS introduced asynchronous telemedicine visits with PCPs in 2013, followed by synchronous telephone visits and video consultations at the end of 2018. Telemedicine visits seamlessly integrated into PCPs’ daily routines. Implementation of telemedicine, complying with guidelines from the Israeli Health Ministry, was gradual, but accelerated during the pandemic’s onset. Patients could choose their preferred visit type without constraints. Telemedicine visits were permissible only after at least one FTF visit in the preceding 12 months as per LHS policy. Reimbursement methods for visits remained consistent, with no fee disparity between FTF and telemedicine visits.

*Study period*: The study spanned the calendar years 2019 through 2023.

*Participants*: This study included all individuals who were members of LHS throughout the study period and had engaged in at least one consultation with a PCP between 2019 and 2023.

*Data sources*: The data for this study were extracted from LHS’s centralized computer database. All interactions with PCPs were fully digitized, and information from electronic medical records was obtained from a central repository.

*Measurement*: Diagnoses were categorized based on the assigned ICD-9 code, utilizing the International Classification of Diseases (ICD) maintained by the World Health Organization (WHO) [17], as detailed in the Appendix. The study focused on the top 21 disease groups exhibiting the highest incidence, excluding COVID-19-related diagnoses due to the unique and evolving standards associated with this new disease.

The duration of each visit and the AADT were calculated in minutes for each visit, corresponding to each ICD-9 code. The change in AADT for each ICD-9 code between 2019 and 2023 was then calculated, with visits lasting less than one minute eliminated. In cases where more than one ICD-9 code was documented for a visit, the visit duration was evenly distributed among the number of diagnoses. The electronic medical record system ensures precision in data collection, as doctors are obligated to access the patient’s electronic record at the start of any visit modality to review personal data, medical history, and document encounter specifics. The termination of a consultation is definitively recorded when the doctor completes their entries and closes the patient’s record, an essential action before addressing any further responsibilities.

Additional patient data encompassed age, sex, and socioeconomic status (SES), the latter defined on a scale of 1 (lowest) to 20 (highest) per Israel’s Central Bureau of Statistics, utilizing the socioeconomic characterization of 1629 geographical units in the country.

*Statistical methods*: Data extraction involved de-identification, facilitated by Python and SQL software. Standard descriptive statistics were employed to depict the data distribution. The annual duration of visits, measured in minutes, was treated as a continuous parameter. All analyses were conducted using R statistical software.

*Patient and public partnership:* In this retrospective study, we analyzed medical record data from a substantial patient population. Our research question centers around the temporal evolution of morbidity distribution and its correlation with primary care physicians’ time investment in patient care. While direct patient participation during the research was precluded due to data access limitations, it is imperative to deliberate involving patients in assessing the significance of our findings and devising strategies to ensure that healthcare quality and accessibility align with their expectations.

## Results

Table 1 shows the age, sex, and SES of the study population between 2019 and 2023.

**Table 1:** Characteristics of the study population

		2019	2020	2021	2022	2023
No. of patients		513,621 (72.63%)	497,227 (71.14%)	515,183 (73.82%)	510,294 (72.47%)	515,076
(% of all patients)						(71.23%
Sex	Female	263,693 (51.34%)	254,184 (51.12%)	263,201 (51.08%)	262,138 (51.37%)	263,326 (51.12%)
	Male	249,890 (48.65%)	243,000 (48.87%)	251,944 (48.90%)	248,141 (48.62%)	251,738 (48.87%)
Mean age (± SD)		37.2 ± 22.7	38.2 ± 22.5	37.8 ± 22.7	38.1 ± 22.8	38.2 ± 22.9
Age bracket	0–2	22,971	18,884	21,066	20,424	20,371
		(4.47%)	(3.80%)	(4.09%)	(4.00%)	(3.95%)
	3–9	41,939	36,874	40,682	40,556	40,717
		(8.17%)	(7.42%)	(7.90%)	(7.95%)	(7.91%)
	10–18	56,276	51,128	54,284	51,999	52,608
		(10.96%)	(10.28%)	(10.54%)	(10.19%)	(10.21%)
	19–29	95,796	95,187	97,113	95,127	95,544
		(18.65%)	(19.14%)	(18.85%)	(18.64%)	(18.55%)
	30–39	72,118	71,266	73,638	72,979	74,189
		(14.04%)	(14.33%)	(14.29%)	(14.30%)	(14.40%)
	40–49	62,892	61,749	62,630	61,983	62,289
		(12.24%)	(12.42%)	(12.16%)	(12.15%)	(12.09%)
	50–59	59,880	58,772	59,444	58,756	59,064
		(11.66%)	(11.82%)	(11.54%)	(11.51%)	(11.47%)
	60–69	53,282	53,285	54,013	54,116	54,121
		(10.37%)	(10.72%)	(10.48%)	(10.60%)	(10.51%)
	70–79	30,346	31,813	33,730	35,963	38,069
		(5.91%)	(6.40%)	(6.55%)	(7.05%)	(7.39%)
	80–89	14,941	14,936	15,234	15,009	14,744
		(2.91%)	(3.00%)	(2.96%)	(2.94%)	(2.86%)
	Others	3,180	3,333	3,349	3,382	3,360
		(0.62%)	(0.67%)	(0.65%)	(0.66%)	(0.65%)
Mean socio-economic status (± SD)		8.09 ± 3.67	8.04 ± 3.67	8.00 ± 3.64	8.00 ± 3.62	7.96 ± 3.61
SES category	0–3	42,217 (9.05%)	42,767 (9.51%)	44,313 (9.55%)	43,497 (9.49%)	53,336 (10.4%)
	4–6	131,053 (28.10%)	126,598 (28.15%)	130,532 (28.14%)	126,887 (27.69%)	45,280 (9.81%)
	7–9	118,870 (25.49%)	115,401 (25.66%)	122,137 (26.33%)	123,798 (27.02%)	126,912 (27.49%)
	10–11	88,287 (18.92%)	83,532 (18.57%)	85,319 (18.39%)	83,968 (18.32%)	84,413 (18.28%)
	12–14	64,775 (13.88%)	61,719 (13.72%)	61,955 (13.35%)	60,907 (13.29%)	60,079 (13.01%)
	15–	21,424 (4.59%)	19,964 (4.44%)	19,848 (4.28%)	19,384 (4.23%)	18,906 (4.09%)
	missing	47,024 (9.16%)	47,326 (9.52%)	51,142 (9.93%)	51,896 (10.17%)	53,336 (10.35%)

SD, standard deviation; SES, socio-economic status

Approximately 70% of the population consults PCPs annually, and our data indicates that this population’s demographics remain consistent across successive years.

Table 2 shows the rate of visits wherein each diagnosis was specified and categorized into distinct diagnostic groups, along with the share of the AADT accrued within each respective diagnosis category.

**Table 2:** Annual visit rate and AADT share for each diagnostic category

Diagnostic categories	Share of visits from all visits that year					Share of the accumulated annual duration of time spent from the total annual time				
	2019	2020	2021	2022	2023	2019	2020	2021	2022	2023
Members approaching health services without reported diagnosis	24.25%	28.19%	29.26%	29.11%	29.66%	18.03%	21.14%	21.46%	21.86%	21.77%
Symptoms	10.05%	9.30%	9.25%	9.19%	9.02%	13.00%	12.46%	12.64%	12.47%	12.37%
Acute respiratory infections	5.54%	3.19%	3.48%	3.59%	3.66%	6.36%	3.73%	4.21%	4.28%	4.41%
Dorsopathies	3.87%	3.52%	3.49%	3.54%	3.58%	4.02%	3.81%	3.84%	3.79%	3.89%
Diabetes mellitus	3.62%	3.76%	3.56%	3.66%	3.58%	3.49%	3.69%	3.54%	3.54%	3.38%
Metabolic and immune disorders	2.97%	2.97%	2.95%	3.21%	3.05%	2.55%	2.68%	2.77%	3.04%	2.83%
Rheumatism, excluding the back	2.21%	2.05%	2.05%	2.09%	2.36%	2.83%	2.74%	2.76%	2.74%	3.11%
Hypertension	3.35%	3.10%	2.58%	2.37%	2.19%	3.13%	2.88%	2.44%	2.26%	2.11%
Nonpsychotic mental disorders	2.73%	2.71%	2.57%	2.54%	2.74%	2.39%	2.37%	2.26%	2.22%	2.43%
Arthropathies and related disorders	1.69%	1.55%	1.51%	1.46%	1.35%	2.02%	1.91%	1.91%	1.84%	1.70%
Diseases of the ear and mastoid process	1.54%	1.20%	1.24%	1.29%	1.38%	1.86%	1.54%	1.66%	1.71%	1.85%
Viral or bacterial diseases—other	1.82%	1.26%	1.24%	1.49%	1.55%	1.94%	1.44%	1.43%	1.65%	1.74%
Diseases of intestines and peritoneum	1.19%	1.18%	1.14%	1.12%	1.14%	1.45%	1.50%	1.48%	1.43%	1.46%
Chronic obstructive pulmonary disease and allied conditions	1.41%	1.09%	1.05%	1.03%	1.06%	1.71%	1.24%	1.28%	1.28%	1.34%
Disorders of the eye and adnexa	1.43%	1.15%	1.14%	1.15%	1.18%	1.47%	1.26%	1.24%	1.25%	1.29%
Contusions	1.32%	1.04%	1.00%	1.02%	1.02%	1.48%	1.25%	1.20%	1.22%	1.21%
Urinary system diseases	1.18%	1.06%	1.02%	1.01%	1.01%	1.31%	1.22%	1.19%	1.18%	1.20%
Non-ischemic heart diseases	0.79%	0.72%	0.69%	0.70%	0.69%	0.93%	0.89%	0.87%	0.86%	0.88%
Screening	0.76%	0.83%	0.81%	0.70%	0.83%	0.63%	0.71%	0.68%	0.57%	0.65%
Ischemic heart disease	0.52%	0.48%	0.45%	0.41%	0.39%	0.58%	0.58%	0.55%	0.51%	0.49%
STDs and viral hepatitis	0.14%	0.12%	0.08%	0.06%	0.06%	0.15%	0.13%	0.09%	0.07%	0.07%

STD, sexually transmitted disease

Visits by individuals approaching health services without reported diagnosis were classified as administrative encounters. These visits typically involved the completion of forms or other paperwork—such as insurance physicals, return-to-work certificates, or court-mandated evaluations—as well as the fulfillment of prescriptions for chronic medications (see Online Appendix 1). Henceforth, these will be referred to as Administrative Visits. They accounted for approximately 28% of all primary care physician (PCP) visits, were of brief duration (mean: 3.3 min), and represented a relatively small proportion of the average annual doctor time (AADT), comprising only 20%.

Symptom-related visits, with prevalent manifestations including fatigue, fever, headache, sore throat, and cough (see Appendix), accounting for roughly 10% of visits, exhibit the longest duration at 5.5 min, with an AADT share of 13%. Despite being the third most prevalent diagnosis, upper respiratory tract infection visits occur at a rate half of that of symptom-related visits, suggesting a disparity in recorded diagnoses and presenting symptoms.

The remaining PCP visits show an even distribution across acute and chronic conditions, with preventative medicine visits comprising less than 1% of the total.

Table 3 shows the annual variations in visit numbers and AADT rates for each diagnostic group relative to the baseline year, 2019.

**Table 3:**
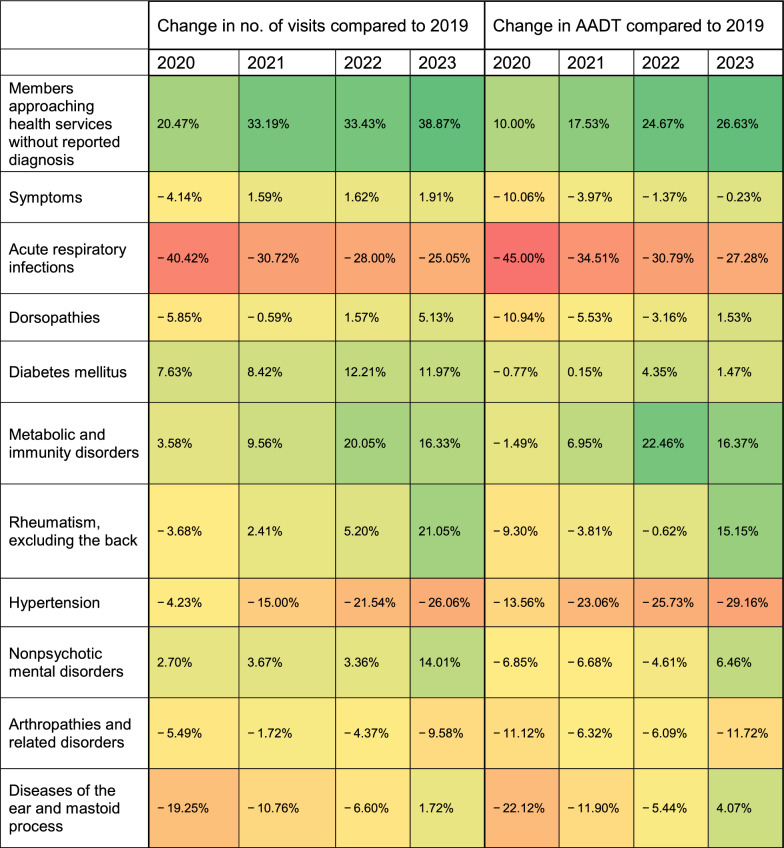

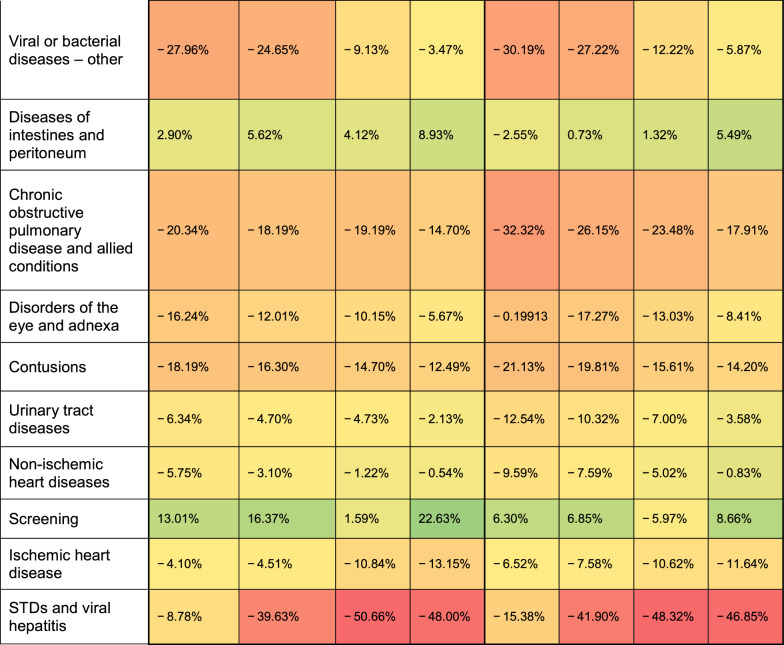
The rate of change in the number of visits and AADT each year, for each diagnosis, compared to 2019*

^*^The range of change is expressed in a color scale between green and red with, the darkest green indicating the greatest increase, and the darkest red indicating the greatest decrease

The most notable change observed is a consistent increase in administrative visits, culminating in an approximate 39% rise in 2023 compared to 2019. Concurrently, the average AADT also exhibited a steady increase, albeit at a lower rate, reaching nearly 27% of the total time spent by physicians in 2023. Another notable shift is in the decline in respiratory tract infection visits, with a substantial reduction in the number of visits and in the corresponding AADT in 2020 compared to the previous year. This reduction moderates somewhat in subsequent years.

Notable decline is observed in the diagnosis of STDs, intensifying over the study period. Until 2022, similar but less pronounced decreases were observed in other disease groups influenced by infectious diseases, such as ear infections, eye infections, and urinary tract infections. However, in 2023, the incidence rates of these diseases increased, approaching the levels observed in 2019. Additionally, there is a discernible decrease in both the number of visits and associated AADT for complaints related to various injuries, particularly limb and head injuries.

In chronic diseases, there was a consistent upward trend in metabolic diseases, particularly obesity. Despite a slight decrease in complaints of large joint pain in 2020, subsequent years showed an increase in prevalence and AADT, especially in 2023. While visits for mental health issues saw a slight increase from 2020 to 2022, the AADT for these visits decreased, indicating shorter appointment durations despite a rise in the number of visits. However, 2023 saw a significant increase in the number of mental health visits, particularly in the last quarter of the year, with an 18% increase in visits and a 20% increase in AADT compared to the average of the first three quarters of 2023.

Unexpectedly, there was a notable increase in visits for preventative medicine, with a more moderate rise in AADT, particularly prominent in 2020–2021 and again in 2023.

## Discussion

Our study examines the changes in medical visit reasons and their effect on PCPs during the COVID-19 pandemic (2020–2023 compared to 2019). To our knowledge, this is the first study to investigate how the pandemic has affected the allocation of PCPs’ time across various diagnoses.

We focused on the 21 most prevalent diagnosis groups, excluding COVID-related diagnoses, to understand how other conditions were influenced by broader pandemic-induced changes in the healthcare system.

### Main finding of this study

Our study finds that the top five visit reasons imposing the highest burden on PCPs remained consistent throughout the pandemic period. These reasons include administrative visits (approximately 21% of all AADT), visits for upper respiratory tract symptoms and infections (around 17%), musculoskeletal pain (about 4%), and diabetes (approximately 3.5%). Although administrative visits constitute nearly 30% of all visits, their shorter duration results in a relatively smaller workload. Conversely, visits related to symptoms, particularly upper respiratory tract symptoms, represented only 10% of visits but imposed a significant burden on physicians in terms of AADT. Additionally, there were no significant differences observed in other disease groups concerning visit rate and their relative contribution to the overall burden in AADT terms.

The rate of administrative visits has been steadily increasing since the onset of the COVID-19 pandemic, reaching nearly 27% of the total time in 2023. Although the AADT generated by these visits is relatively low, it is noteworthy that it has been increasing annually and likely has not yet reached its peak. This trend may be attributed to the shift in the mix of visit types and the rise in telemedicine visits, particularly for prescription renewals, approvals, and referrals for routine follow-ups [18]. More time is needed to evaluate the effect of telemedicine on the time invested by primary physicians because the adaptation to telemedicine may align the content of telemedicine visits more closely with that of in-person visits [5, 19].

In terms of mental health issues, there was a noticeable increase in number of visits in 2021 and 2022, a trend supported by previous studies, although not consistently [8, 20, 21]. As the pandemic wanes, these visits have become shorter and, at least until 2022, have resulted in less cumulative burden on PCPs. Further investigation is needed to explore potential associations with the increased rate of telemedicine visits, ascertain whether there was an increased burden on mental health caregivers, and assess whether the shorter primary care visits have affected overall quality of care. Understanding the quality of the response to mental health issues is crucial, as the stress, anxiety, and loneliness prevalent during pandemic peaks can have far-reaching effects on various other morbidities [22, 23]. In 2023, there was even more notable increase in the number of visits for mental health problems, particularly in the last quarter of the year. This trend likely reflects patients’ responses to the brutal attack that occurred in October and the subsequent war.

Across all disease groups involving exposure to infections, there was a consistent decrease in the burden on PCPs that moderated over time. For example, respiratory tract infections declined by up to 45% in 2020, gradually increasing but remaining approximately 27% lower in 2023 compared to 2019. A similar, though less pronounced, trend was observed in other viral and bacterial infections, as well as ear, eye, intestinal, and urinary tract diseases. Notably, STDs saw a significant downward trend, with nearly a 50% reduction in 2023 compared to 2019. While this trend is encouraging, it should be interpreted with caution due to the relatively low prevalence of STDs in the general population, which may amplify the impact of small absolute changes.

### What is already known on this topic

The decrease in the rate of infectious diseases is attributed to reduced social contact and infection prevention measures implemented during the pandemic [24–26].

Beginning in the second half of 2021, with the easing of restrictions, there was an increase in respiratory tract infections, particularly due to Respiratory Syncytial Virus and rhinovirus, especially in children during late spring months [27–29]. Variation in bacterial diseases is observed depending on the type of bacteria and geographic region, with most areas reporting seasonal increases. However, from an annual perspective, lower morbidity was maintained in 2022 from pre-pandemic levels.

Regarding STDs, the available information is not consistent, with some countries reporting similar findings [30] while others report the opposite [31, 32]. Identical to other infectious diseases, underreporting and underdiagnosis were noted due to reluctance to visit clinics [33].

The decline in injuries and musculoskeletal pain is likely attributed to reduced physical activity, decreased exposure to traffic accidents, and a decline in sports and leisure activities [23]. Across various conditions, this trend aligns with periods of social restrictions, gradually increasing upon restrictions’ relaxation. However, even in 2023, the burden remains relatively low for injuries, warranting continued observation, particularly considering reports of sports injuries due to reduced training which persisted throughout the pandemic across its various waves [34].

Changes in lifestyle may contribute to the increased burden caused by chronic diseases, particularly metabolic abnormalities. Evidence from our research and reports of rising obesity indices and blood lipid values at the end of 2021 [35] further support this observation, even after a return to previous lifestyle became feasible. The surge in obesity diagnosis may also be attributed, in part, to the introduction of new weight loss drugs and increased public awareness.

The number of visits for diabetes treatment is gradually increasing, with a significant additional burden noted in 2022. In 2023, there is a certain trend of moderation in the burden, and more time is needed to determine if this trend is consistent. Global studies indicate a slight rise in diabetes follow-ups, possibly due to visits shifting to telemedicine [36], which improves accessibility without compromising quality. Evidence also suggests a link between COVID-19 infection and the onset of diabetes [37, 38].

Conversely, there was a discernible decrease in visits for treating hypertension and ischemic heart disease, a trend observed globally at the pandemic’s onset [39–41]. Avoiding clinic visits may adversely affect care, compounded by an increase in other risk factors and the independent risk of COVID-19 infection for hypertension [42] and ischemic heart disease [43]. This finding underscores the need for attention and proactive measures to mitigate future complications.

In LHS, akin to global trends, there was a noticeable decrease in preventative medicine-related activities [8, 44]. However, we observed an increase in the number of visits for early detection of breast cancer and screening for abdominal aortic aneurysm, attributed to a proactive organizational effort to encourage these screening measures in 2020–2021 and in 2023 to address decreased screenings at the beginning of the pandemic. Despite the rise in visits, they did not create a significant AADT burden as they were relatively short.

### Strengths of this study

- Comprehensive examination of the overall distribution of diagnoses handled by PCPs
- Coverage of a valuable time frame as the pandemic diminishes and people return to pre-pandemic healthcare and lifestyle practices
- Time-based assessment to gauge the burden on PCPs, offering a more accurate reflection of resource requirements
- The methodology employed in our study facilitates the identification of potentially under-addressed conditions and provides insights for optimizing resource allocation. This is particularly relevant in scenarios where redirecting specific conditions to telemedicine leads to shorter visit durations [45, 46] or when there is an increase in the complexity of certain complaints [47].
- Congruence between study population demographics and previous investigations [12, 48], adding validity to findings.
- Consistent policies governing service provision and reimbursement across all types of visits throughout the study period, mitigating potential biases.
- Utilization of ICD-9 codes with recognized moderate-to-high Positive Predictive Value (PPV) and Negative Predictive Value (NPV) (≥ 70%) [49]. This acknowledged limitation in predictive value is well-accepted in the literature [50] and remained consistent over the study years, ensuring the reliability of comparisons over time.
- Although Leumit serves a slightly younger demographic and has a somewhat greater presence in peripheral regions compared to other HMOs, previous analyses have shown no significant differences in the distribution of chronic diseases between Leumit and the broader Israeli population. These similarities enhance the external validity of our findings and support their generalizability to other Israeli HMOs, thereby reinforcing the study’s relevance for national healthcare policy and practice.

### Limitations of this study

- Caution is warranted when extrapolating burden trends to actual morbidity trends, as the encouragement of self-conservative treatment during the pandemic may have influenced patient behavior regarding non-COVID diseases.
- This study specifically assessed diseases’ burden on PCPs and did not address unreported morbidity, thereby limiting generalizability.
- Evaluation of the distribution of FTF visits and various types of telemedicine within each diagnosis group is not addressed, requiring further research.
- Analyzing data over the course of a year may not effectively distinguish the specific effects of lifting infection prevention measures on morbidity trends, as these measures were progressively altered over the year of the study.
- During the last three months of 2023, the patient population was exposed to the war, which could potentially bias the findings. However, given that this period is relatively short and that the trends, except in the field of mental health, have remained consistent, it is reasonable to assume that the impact has not yet significantly altered the characteristics of patient visits.
- Certain types of morbidity may demonstrate a cumulative effect over time, a phenomenon that our current research has not yet been able to demonstrate. Continued observation is necessary to further investigate this aspect.
- In this study, each diagnosis and each clinical visit were treated with equal analytical weight. This methodological choice does not reflect the real-world variability that exists among patients with the same diagnosis, nor the potential differences between multiple visits by the same patient. Such simplification may limit the granularity and applicability of the findings. In contexts where precise evaluation of physician time investment per diagnosis is critical for decision-making, future studies should be designed to incorporate methodologies capable of capturing and quantifying this variability.

## Conclusions and recommendations

1. This study highlights critical imbalances in how PCPs allocate their time, revealing systemic inefficiencies that warrant immediate policy attention. The continuous rise in the burden of administrative tasks—now approaching 30% of all visits—calls for a comprehensive assessment of how PCP time is utilized. Policymakers should evaluate the impact of organizational changes and public health directives on clinical workflows and consider strategies such as digital automation and task delegation to reduce bureaucratic overhead and enhance system efficiency.
2. Evaluation of PCP time distribution compared to optimal distribution, identifying areas of underutilization (e.g., hypertension and ischemic heart disease monitoring, certain preventative medicine aspects) and conditions with increased morbidity (e.g., obesity and hyperlipidemia). Intervention plans should aim to ensure optimal follow-up, early detection, and prevention of future morbidity. Understanding desirable changes, such as decreased pain complaints, is crucial for preserving their beneficial effects.
3. The integration of telemedicine into primary care presents both opportunities and challenges. While it has the potential to alleviate in-person visit loads, its implementation must be re-evaluated to ensure equitable access, clinical appropriateness, and alignment with provider capacity. Special attention should be given to vulnerable populations who may face barriers to digital health services.
4. The pandemic’s indirect effect on reducing infectious disease burden—likely due to behavioral and hygiene adaptations—suggests that continued public education on infection control could yield long-term health benefits. Sustaining these practices may help mitigate future outbreaks and reduce the burden on primary care.
5. To operationalize these insights, we recommend the development of standardized triage protocols to prioritize high-complexity cases, updated telemedicine guidelines that promote equitable and effective virtual care, and streamlined referral and documentation processes. These reforms should be equity-focused, ensuring that system improvements do not inadvertently disadvantage patients with limited digital literacy or access. Collectively, these measures can help recalibrate primary care delivery to be more resilient, efficient, and responsive to evolving population needs.

## Supplementary Information

Supplementary Material 1.

## Data Availability

The datasets analyzed during the current study are not publicly available as they are business information but are available from the corresponding author upon reasonable request.
